# Structural Mechanism Underpinning Cross-reactivity of a CD8^+^ T-cell Clone That Recognizes a Peptide Derived from Human Telomerase Reverse Transcriptase*

**DOI:** 10.1074/jbc.M116.741603

**Published:** 2016-11-30

**Authors:** David K. Cole, Hugo A. van den Berg, Angharad Lloyd, Michael D. Crowther, Konrad Beck, Julia Ekeruche-Makinde, John J. Miles, Anna M. Bulek, Garry Dolton, Andrea J. Schauenburg, Aaron Wall, Anna Fuller, Mathew Clement, Bruno Laugel, Pierre J. Rizkallah, Linda Wooldridge, Andrew K. Sewell

**Affiliations:** From the ‡ Division of Infection and Immunity and Systems Immunity Research Institute, Cardiff University School of Medicine, Heath Park, Cardiff CF14 4XN, United Kingdom,; the § Mathematics Institute, University of Warwick, Coventry CV4 7AL, United Kingdom,; the ¶ Cardiff University School of Dentistry, Heath Park, Cardiff CF14 4XY, United Kingdom,; the ‖ Queensland Institute of Medical Research Berghofer Medical Research Institute, Brisbane, Queensland 4029, Australia,; ** James Cook University, Cairns, Queensland 4870, Australia, and; the ‡‡ Faculty of Health Sciences, University of Bristol, Bristol BS8 1TD, United Kingdom

**Keywords:** peptides, surface plasmon resonance (SPR), telomerase, tumor immunology, X-ray crystallography, T cell receptor, T cell degeneracy, T cells

## Abstract

T-cell cross-reactivity is essential for effective immune surveillance but has also been implicated as a pathway to autoimmunity. Previous studies have demonstrated that T-cell receptors (TCRs) that focus on a minimal motif within the peptide are able to facilitate a high level of T-cell cross-reactivity. However, the structural database shows that most TCRs exhibit less focused antigen binding involving contact with more peptide residues. To further explore the structural features that allow the clonally expressed TCR to functionally engage with multiple peptide-major histocompatibility complexes (pMHCs), we examined the ILA1 CD8^+^ T-cell clone that responds to a peptide sequence derived from human telomerase reverse transcriptase. The ILA1 TCR contacted its pMHC with a broad peptide binding footprint encompassing spatially distant peptide residues. Despite the lack of focused TCR-peptide binding, the ILA1 T-cell clone was still cross-reactive. Overall, the TCR-peptide contacts apparent in the structure correlated well with the level of degeneracy at different peptide positions. Thus, the ILA1 TCR was less tolerant of changes at peptide residues that were at, or adjacent to, key contact sites. This study provides new insights into the molecular mechanisms that control T-cell cross-reactivity with important implications for pathogen surveillance, autoimmunity, and transplant rejection.

## Introduction

Recognition of peptide-major histocompatibility complexes (pMHCs)^5^ by the clonally expressed αβ T-cell receptor (TCR) mediates T-cell immunity. Although TCRs generally interact with pMHC via a conserved binding mode, with the TCRα chain positioned over the MHCα1 domain and the TCRβ chain positioned over the MHCα2 domain, the TCR complementarity-determining region (CDR) loops can use a variety of mechanisms to probe both the MHC surface and bound peptide (1). This flexible binding probably mediates the ability of a single TCR to interact productively with a large range of different epitopes (2–6). Thus, TCR degeneracy enables the approximately 25 million distinct TCR clonotypes expressed by an individual host (7) to have the potential to recognize the entire theoretical peptide universe that could be presented by MHC (2, 8), minimizing the likelihood of pathogens escaping immune surveillance. Given the highly diverse number of TCR-pMHC binding modes seen to date, it is reasonable to predict that different TCRs will exhibit distinct levels of cross-reactivity, depending on the chemical characteristics of their CDR loops and how they interact with pMHC. Such distinctions could determine whether certain TCRs are more likely to offer sufficient protection against hypervariable pathogens, such as human immunodeficiency virus type 1, hepatitis B virus, hepatitis C virus, and influenza, or conversely to trigger autoimmune disease.

Although new quantitative information on the extent of T-cell cross-reactivity has recently come to light (3, 5, 9), the molecular rules that determine this important facet of cellular adaptive immunity remain unclear. Understanding TCR binding degeneracy, and the ensuing T-cell cross-reactivity it enables, is of emerging importance given the increasing use of T-cell therapies using modified TCRs, one of which has already demonstrated the dangers of unintentional T-cell cross-reactivity with self-ligands (10–12). Currently, there are few examples of TCR-pMHC complex structures for which the cross-reactivity profiles of the corresponding T-cell clone have also been determined (3, 13, 14). In a previous study, we demonstrated that an insulin-reactive human CD8^+^ T-cell clone (1E6) could recognize upward of one million unique peptide ligands. The structure of the 1E6 TCR with its cognate ligand revealed focused TCR-peptide binding with the interaction of only two TCR residues and two adjacent peptide residues accounting for the majority of the binding interface. We speculated that this focused binding might enable the 1E6 TCR to tolerate changes outside of the core motif, mediating the high level of degeneracy. In support of this, another study recently demonstrated that a high level of cross-reactivity was mediated by similar focused TCR-peptide binding by an MHC class II-restricted TCR (13). However, whether TCRs must exhibit focused peptide binding to cross-react remains unclear. This is an important question because, unlike the two examples of focused TCR-peptide binding mentioned above, most TCRs that have been studied structurally to date make more comprehensive interactions with the pMHC surface. Thus, whether a “typical” TCR binding footprint can underpin T-cell cross-reactivity remains unknown.

Here, we used a well characterized CD8^+^ T-cell clone (ILA1) (15) that responds to residues 540–548 (sequence, ILAKFLHWL) of human telomerase reverse transcriptase to further investigate the structural basis of TCR degeneracy. We have previously characterized a limited number of altered peptide ligands (APLs) for the ILA1 T-cell clone that exhibit different potencies in terms of T-cell activation (9, 15), corresponding to a wide range of binding affinities with the ILA1 TCR (15–17). These previous findings clearly demonstrate that the ILA1 T-cell clone can recognize multiple different peptide ligands. Here, we solved the structure of the ILA1 TCR in complex with HLA-A*0201-ILAKFLHWL (A2-ILA) and several previously defined APLs. Combined with biophysical analysis, we demonstrate the molecular mechanism for antigen discrimination by the ILA1 TCR and model the mode of cross-reactivity with these APLs. In addition, we used our previously published peptide sampling approach (3) to estimate the number of pMHCI molecules that could be recognized by the ILA1 TCR. These data offer novel insight into the molecular factors that determine T-cell cross-reactivity, extending our understanding of the nature of T-cell antigen discrimination.

## Results

### 

#### 

##### The ILA1 TCR Makes a Broad Contact Network with A2-ILA

**FIGURE 1.**
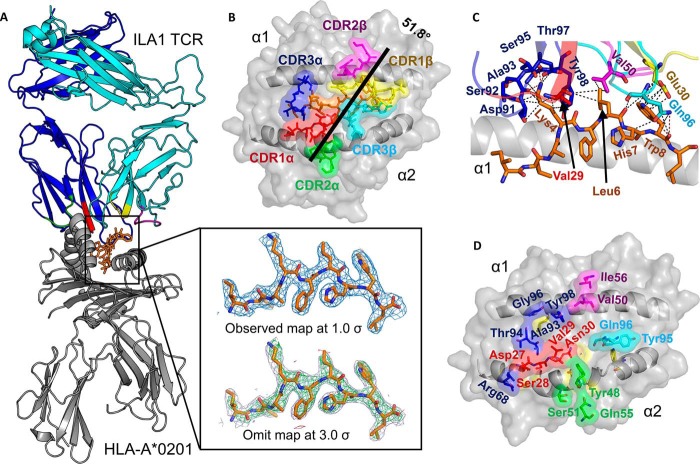
**The ILA1 TCR uses a broad binding footprint to engage A2-ILA.** *A*, the overall binding mode of ILA1 TCR (*blue* and *cyan* schematic; CDR loops shown in *multicolored* schematic) in complex with A2-ILA (*gray* schematic and *orange sticks*). The *box* shows the observed map (*top*) at 1.0 σ and an omit maps (*below*) in which the model was refined in the absence of the ILA peptide with difference density contoured at 3.0 σ; positive contours are shown in *green*, and negative contours are shown in *red. B*, position of the ILA1 TCR CDR loops (*multicolored sticks*) with the ILA peptide (*orange sticks*) is shown in the HLA-A*0201 binding groove (*gray surface*). The crossing angle of the ILA1 TCR (*black line*) was calculated using previously published parameters (37). Briefly, this crossing angle represents the angle between a best fit straight line through the Cα atoms from the two MHC helices and a line that links the disulfide bond in the TCR α-chain variable region to the disulfide bond in the TCR β-chain variable region. *C*, interaction between residues in the ILA1 TCR CDR loops (*multicolored sticks*) and the ILA1 peptide (*orange sticks*) with the MHC α1 helix shown as a *gray* schematic. *D*, the ILA1 TCR residues in the CDR loops that contact the MHC surface are shown in *multicolored* schematic, and the surface with the MHC binding groove is shown in *gray* schematic and surface. CDR loops are colored as follows throughout: CDR1α, *red*; CDR2α, *green*; CDR3α, *blue*; CDR1β, *yellow*; CDR2β, *purple*; and CDR3β, *cyan*.

We solved the structure of the ILA1 TCR-A2-ILA complex at 2.8 Å in space group P 1 21 1 with crystallographic *R*_work_/*R*_free_ ratios within accepted limits as shown by the theoretically expected distribution (18) (Table 1). The electron density was high quality throughout, represented by an omit map analysis of the ILA peptide (Fig. 1*A*). The ILA1 TCR utilized a canonical binding mode to engage A2-ILA (Fig. 1*A*) with a buried surface area (2507.2 Å^2^) and surface complementarity (TCR-pMHC = 0.641) within the normal range (Table 2) (1). The TCR α-chain was orientated over the MHCI α1 helices, and the TCR β-chain was oriented over the MHCI α2 helices (Fig. 1*B*), positioning the CDR loops of the ILA1 TCR over the central portion of the peptide, enabling contacts with 4 of the 9 peptide residues (Fig. 1*C*). Peptide residues Lys-4 and Trp-8 engaged in a complex network of contacts with the TCR CDR1/3α loops and CDR1/3β loops, respectively, contributing 39 of the 49 total peptide contacts (Table 2). Binding to Lys-4 involved a tight ball-and-socket interaction with 7 TCR residues, whereas contacts with Trp-8 were less restrictive, involving only TCR β-chain residues Glu-30 and Gln-96 (Table 3 and Fig. 1*C*). The 49 TCR interactions with peptide were supported by 58 contacts with MHC, involving 12 TCR α-chain residues and 4 TCR β-chain residues (Fig. 1*D*), contributing to the slightly TCR α-chain-skewed binding mode (α, 58%; β, 42% of total contacts). Notably, the TCR α-chain residue Arg-68 in the framework region loop (outside of the traditional CDR loops) formed two salt bridges with MHC residue Glu-166, and all of the MHC restriction triad residues (Arg-65, Ala-69, and Gln-155) (19) interacted with the ILA1 TCR (Table 3).

**TABLE 1:** **Data collection and structure refinement statistics** Values in parentheses refer to the highest resolution bin. One crystal was used for solving each structure. DLS, Diamond Light Source; r.m.s., root mean square; n/a, not applicable; CC1/2, correlation coefficient.

	ILA1-A2-ILA	A2-ILA3G8R	A2-ILA3G	A2-ILA8T	A2-ILA8E
**Data collection**					
Protein Data Bank code	5MEN	5MEO	5MEP	5MEQ	5MER
Space group	P 1 21 1	P 21 21 21	P 21 21 21	P 1 21 1	P 21 21 21
Beamline	DLS I24	DLS I04	DLS I02	DLS I04	DLS I02
Cell dimensions					
*a* (Å)	93.2	49.2	119.4	53.34	45.7
*b* (Å)	48.7	74.9	169.6	81.44	119.0
*c* (Å)	118.1	125.8	47.1	56.77	170.3
α (°)	90	90	90	90	90
β (°)	108.2	90	90	113.5	90
γ (°)	90	90	90	90	90
Resolution maximum (Å)	2.81 (2.88–2.81)	1.77 (1.82–1.77)	2.71 (2.78–2.71)	2.27 (2.33–2.27)	1.88 (1.93–1.88)
*R*_merge_ (%)	0.100 (0.73)	0.105 (0.718)	0.105 (.831)	0.086 (0.632)	0.098 (0.621)
Total measurements	91,454 (6,913)	332,927 (22,417)	195,005 (13,976)	74,664 (5,741)	545,975 (42,439)
Unique reflections	25,031 (1,865)	46,011 (3,362)	26,894 (1,974)	20,523 (1,527)	76,630 5,603
*I*/σ*I*	9.2 (1.9)	10.5 (2.5)	14.1 (2.3)	12.8 (1.9)	10.8 (3.2)
CC1/2	n/a	n/a	0.997 (0.714)	0.997 (0.842)	0.997 (0.913)
Completeness (%)	99.7 (99.8)	100 (100)	100 (100)	99.3 (98.9)	99.9 (100)
Multiplicity	3.7 (3.7)	7.2 (6.7)	7.3 (7.1)	3.5 (3.8)	7.1 (7.6)

**Refinement**					
Resolution (Å)	56.11–2.81	48.16–1.77	42.39–2.71	48.90–2.27	34.06–1.9
No. reflections in work set	23,666	43,626	25,511	19,484	72,747
No. reflections in *R*_free_ set	1,267	2,319	1,333	1,023	3,797
*R*_work_*/R*_free_ (%)	18.9/27.2	17.1/21.4	18.2/23.9	20.3/26.4	18.4/22.4
Mean B value (Å^2^)	57.1	27.7	52.2	37.7	33.0
Wilson B-factor (Å^2^)	70.5	21.9	39.6	32.97	28.6
Overall coordinate error (Å)	0.377	0.082	0.252	0.232	0.098
r.m.s. deviations					
Bond lengths (Å)	0.017	0.019	0.013	0.019	0.019
Bond Angles (°)	2.025	1.983	1.658	1.976	1.967
Ramachandran plot statistics					
Most favored region (%)	92.27	97.85	97.23	96.54	98.12
Allowed region (%)	6.38	2.15	2.77	3.19	1.88
Outliers (%)	1.35	0	0	0.27	0

**TABLE 2:** **ILA1-A2-ILA contact summary** Buried surface area, 2507.2 Å^2^; surface complementarity for TCR-MHC, 0.635; surface complementarity for TCR-peptide, 0.707; surface complementarity for TCR-pMHC, 0.641; crossing angle, 51.8°, calculated as described previously (37). vdW, van der Waals; FW, framework region.

	vdW (≤4 Å)	H-bonds (≤3.4 Å)	Salt bridges (≤3.4 Å)
MHC	58	6	2
Peptide	49	4	1
Peptide Lys-4	20	2	1
Peptide Trp-8	19	2	0
TCRα	62	7	3
CDR1α	16	2	0
CDR2α/FW	9	2	2
CDR3α	37	3	1
TCRα Asp-97	6	2	0
TCRα Arg-68	4	0	2
TCRβ	45	3	0
CDR1β	13	0	0
CDR2β	7	0	0
CDR3β	25	3	0
TCRβ Gln-96	18	3	0
**Total contacts**	**107**	**10**	**3**

**TABLE 3:** **ILA1-A2-ILA contacts** A 3.4 Å cutoff was used for H-bonds and salt bridges, and a 4 Å cutoff was used for van der Waals (vdW). FW, framework region.

CDR loop	Gene usage	TCR residue	Peptide residue	MHC residue	vdW (≤4 Å)	H-bonds (≤3.4 Å)
CDR1α	*TRAV22*	Asp-27^Oδ2^		Thr-163^Oγ1^	4	1
	*TRAV22*	Asp-27^Oδ1^		Glu-166^Oϵ2^	2	1
	*TRAV22*	Ser-28		Ala-158	1	
	*TRAV22*	Val-29		Gln-155	4	
	*TRAV22*	Val-29	Lys-4		4	
	*TRAV22*	Asn-30		Gln-155	1	
CDR2α	*TRAV22*	Tyr-48		Glu-154	1	
	*TRAV22*	Tyr-48		Gln-155	2	
	*TRAV22*	Ser-51^O^		Glu-154^Oδ^	1	1
	*TRAV22*	Ser-51		Arg-157	1	
	*TRAV22*	Gln-55^Oϵ1^		Glu-154^Oϵ2^		1
FWα	*TRAV22*	Arg-68^NH1/NH2^		Glu-166^Oϵ1/Oϵ2^	4	2 salt bridges
CDR3α	*TRAJ40*	Asp-91^Oδ1^	Lys-4^Nζ^		3	1 salt bridge
	*TRAJ40*	Ser-92^O^	Lys-4^Nζ^		2	1
	*TRAJ40*	Ala-93		Lys-66	1	
	*TRAJ40*	Ala-93		Thr-163	2	
	*TRAJ40*	Ala-93	Lys-4		2	
	*TRAJ40*	Thr-94^O^		Lys-66^Nζ^	3	1
	*TRAJ40*	Thr-94		Trp-167	3	
	*TRAJ40*	Ser-95	Lys-4		4	
	*TRAJ40*	Gly-96		Arg-65	3	
	*TRAJ40*	Thr-97^O^	Lys-4^Nζ^		3	1
	*TRAJ40*	Tyr-98		Lys-66	3	
	*TRAJ40*	Tyr-98		Ala-69	2	
	*TRAJ40*	Tyr-98	Lys-4		2	
	*TRAJ40*	Tyr-98	Leu-6		4	
CDR1β	*TRBV6*	Glu-30	Trp-8		13	
CDR2β	*TRBV6*	Val-50		Val-152	1	
	*TRBV6*	Val-50	Leu-6		1	
	*TRBV6*	Val-50	Trp-8		1	
	*TRBV6*	Ile-54		Gln-72	4	
CDR3β	*TRBJ1-1*	Tyr-95		Lys-146	5	
	*TRBJ1-1*	Tyr-95		Ala-150	2	
	*TRBJ1-1*	Gln-96		Lys-146	2	
	*TRBJ1-1*	Gln-96^Oϵ1^		Trp-147^Nϵ1^	4	1
	*TRBJ1-1*	Gln-96		Ala-150	1	
	*TRBJ1-1*	Gln-96		Val-152	1	
	*TRBJ1-1*	Gln-96	Leu-6		2	
	*TRBJ1-1*	Gln-96	His-7		3	
	*TRBJ1-1*	Gln-96^Oϵ1^	Trp-8^N/O^		5	2

**FIGURE 2.**
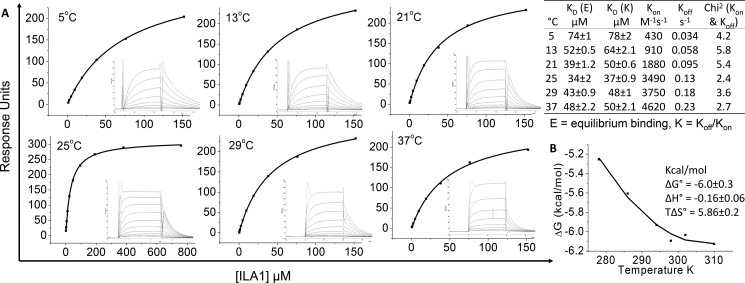
**Thermodynamic analysis of the ILA1 TCR with A2-ILA.** *A*, eight to ten serial dilutions of the ILA1 TCR were injected in duplicate over A2-ILA at 5, 13, 21, 25, 29, and 37 °C. The equilibrium binding constants (*K_D_*) were calculated using a non-linear curve fit (*y* = (P_1_*x*)/P_2_ + *x*)), and kinetic and affinity parameters are shown in the *table. B*, the binding free energies, Δ*G*^0^ (Δ*G*^0^ = *RT* ln *K_D_*), were plotted against temperature (K) using non-linear regression to fit the three-parameter van 't Hoff equation (*RT* ln *K_D_* = Δ*H*^0^ − *T*Δ*S*^0^ + ΔCp^0^(*T* − *T*_0_) − *T*ΔCp^0^ ln(*T*/*T*_0_) with *T*_0_ = 298 K).

We have shown previously that the ILA1 TCR binds with a moderate/weak affinity (*K_D_* = 34 μm) to A2-ILA (20), consistent with the observation that TCRs specific for self-pMHCIs usually bind at the lower end of the TCR-pMHC affinity scale (21, 22). We performed a thermodynamic analysis of the ILA1 TCR-A2-ILA interaction by measuring binding using surface plasmon resonance at a range of temperatures (5–37 °C) (Fig. 2*A*). These analyses demonstrated that the weak affinity was not temperature-dependent, ranging from *K_D_* = 34 (25 °C) to 74 μm (5 °C). At physiological temperature (37 °C), the affinity was in the middle of this range (*K_D_* = 48 μm). The energetic analysis (Fig. 2*B*) revealed that the ILA1 TCR-A2-ILA interaction was driven entropically (*T*Δ*S* = 5.86 kcal/mol) with only a minor change in enthalpy (Δ*H* = −0.16 kcal/mol). These values indicate almost no net loss, or gain, in electrostatic interactions during complex formation, indicative of structural reordering of the TCR and/or pMHC when binding. The entropic contribution suggested that ordered water molecules are squeezed out at the interface as the TCR and pMHC engage. Overall, these analyses demonstrated that the ILA1 TCR utilizes a relatively broad binding footprint, contacting spatially distant regions on the peptide and involving 21 different TCR residues contacting 17 A2-ILA residues (4 peptide and 13 MHC residues), contributing to an entropically driven, moderate-to-weak affinity interaction.

##### APLs Guide ILA1 Antigen Recognition through Structural Alterations in Peptide Conformation

**FIGURE 3.**
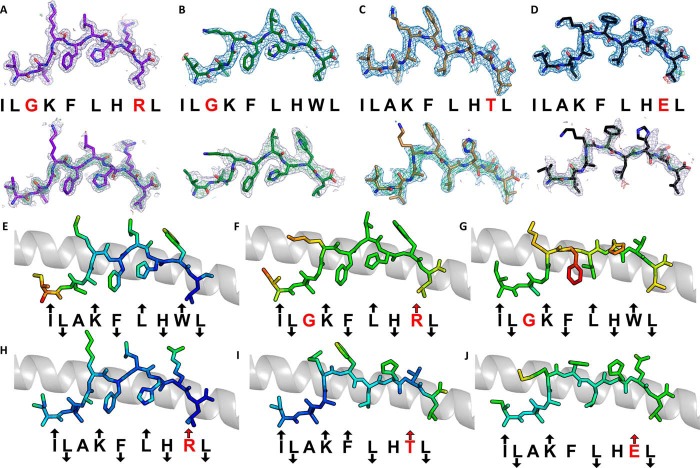
**Density plot, omit map, and B-factor analysis of ILA1 peptide variants.** *A–D*, *top*, the observed map at 1.0 σ is shown. *Bottom*, omit maps are shown in which the model was refined in the absence of the ILA peptide variants with difference density contoured at 3.0 σ; positive contours are shown in *green*, and negative contours are shown in *red. A*, A2-ILGKFLHRL (*purple sticks*). *B*, A2-ILGKFLHWL (*green sticks*). *C*, A2-ILAKFLHTL (*sand sticks*). *D*, A2-ILAKFLHEL (*black sticks*). A2-ILAKFLHRL was solved previously (23). *E–J*, each APL is colored by B-factor with *light blue* representing a low B-factor and *red* representing a high B-factor. The conformation of each APL (*sticks*) with *arrows* indicating the direction of each residue in the peptide (solvent-exposed, MHC anchor, or in between) with the MHC α1 helix shown as a *gray* schematic. Residues in *red* indicate differences from the index sequence. An *up arrow* indicates solvent-exposed, a *down arrow* indicates anchor position, and *no arrow* indicates an intermediate position. *E*, A2-ILAKFLHWL. *F*, A2-ILGKFLHRL. *G*, A2-ILGKFLHWL. *H*, A2-ILAKFLHRL. *I*, A2-ILAKFLHTL. *J*, A2-ILAKFLHEL.

**FIGURE 4.**
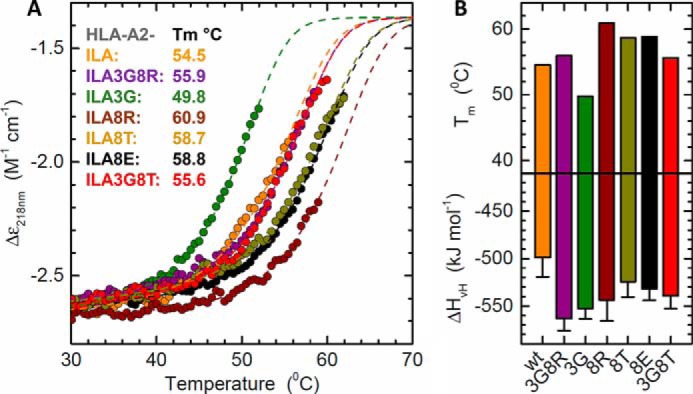
**Stability of HLA-A2-ILA variants using circular dichroism.** *A*, CD thermal denaturation curves recorded at 218 nm are shown for selected peptide-HLA class I samples. *Dots* represent measured values fitted assuming a two-state trimer-to-monomer transition (*dashed lines*) as described under “Experimental Procedures.” *B*, bar graphs of the thermal stability with respect to melting temperature (*upper panel*) and van 't Hoff's enthalpy of unfolding (*lower panel*). *Error bars* represent S.D. resulting from the multivariable curve fitting.

**FIGURE 5.**
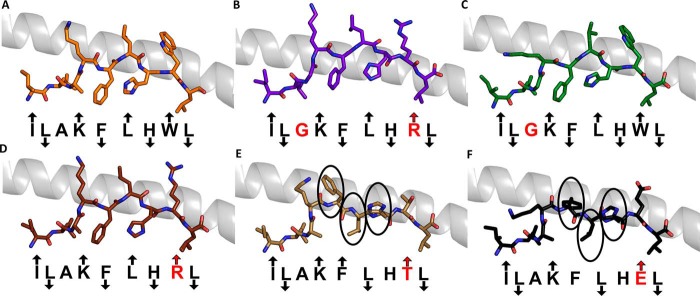
**Structural analysis of ILA1 TCR ligands.** Shown is the conformation of each APL (*sticks*) demonstrating the direction of each residue in the peptide (solvent-exposed, MHC anchor, or in between) with the MHC α1 helix shown as a *gray* schematic. Residues in *red* indicate differences from the index sequence. An *up arrow* indicates solvent-exposed, a *down arrow* indicates anchor position, and *no arrow* indicates an intermediate position. *A*, A2-ILAKFLHWL (*orange sticks*). *B*, A2-ILGKFLHRL (*purple sticks*). *C*, A2-ILGKFLHWL (*green sticks*). *D*, A2-ILAKFLHRL (*brown sticks*) (reproduced from Ref. 23). *E*, A2-ILAKFLHTL (*sand sticks*). *F*, A2-ILAKFLHEL (*black sticks*). The *circled* residues in *E* and *F* face in different directions as compared with the index telomerase sequence (ILAKFLHWL) in *A*.

We have previously characterized a number of APLs that alter the T-cell activation profile and TCR binding affinity of the ILA1 T-cell clone (9, 15, 16). To investigate how these ligands adjust TCR interactions to tune affinity, we solved the structure of four APLs (A2-ILA3G8R, IL**G**KFLH**R**L; A2-ILA3G, IL**G**KFLHWL; A2-ILA8T,ILAKFLH**T**L; and A2-ILA8E, ILAKFLH**E**L), included our previously published APL structure (A2-ILA8R, ILAKFLH**R**L) (23), and used the ILA1-A2-ILA complex as a model. The electron density was high quality throughout, represented by an omit map analysis of the ILA peptide variants (Fig. 3, *A–D*), and B-factor analysis indicated that there were no major differences in peptide mobility across the peptide variants (Fig. 3, *E–J*). Thermal stability analysis demonstrated that most of the APLs had a similar apparent *T_m_* value (the term “apparent *T_m_*” is used here because the protein irreversibly aggregates at high temperature) of around 55 °C with extremes in the range of 50 to 61 °C (Fig. 4). These similar stabilities are consistent with our previous observation that all these APLs bind equally to HLA-A2 on the cell surface (15). As we have shown previously in other systems (24), apparent *T_m_* values correlated poorly with antigen potency (for instance, A2-ILA3G had the lowest apparent *T_m_* value but was a potent activator of the ILA1 T-cell clone), suggesting that different pMHC cell surface expression levels were a minor factor in T-cell recognition. The A2-ILA3G structure was determined at 2.7 Å resolution, and the other APL structures were determined at resolutions between at 1.9 and 1.8 Å with crystallographic *R*_work_/*R*_free_ ratios within accepted limits as shown by the theoretically expected distribution (18) (Table 1). The overall conformations of A2-ILA3G8R, A2-ILA3G, and A2-ILA8R were virtually identical to A2-ILA (Fig. 5, *A–D*) with Lys-4, Leu-6, and Trp-8 pointing up and away from the MHC binding groove and Leu-2, Phe-5, His-7, and Leu-9 acting as primary and secondary anchors, indicating that a molecular mimicry mechanism underpins ILA1 TCR recognition of these APLs. In contrast, in the A2-ILA8T and A2-ILA8E structures, peptide residues 5–7 were flipped so that Leu-6 acted as a secondary anchor and Phe-5 and His-7 were in more solvent-exposed positions (Fig. 5, *E* and *F*). In both peptide variants, the mutated residue was at position 8, distal from this structural rearrangement. Closer inspection of the structures did not reveal an obvious mechanism for this indirect effect on peptide conformation.

**FIGURE 6.**
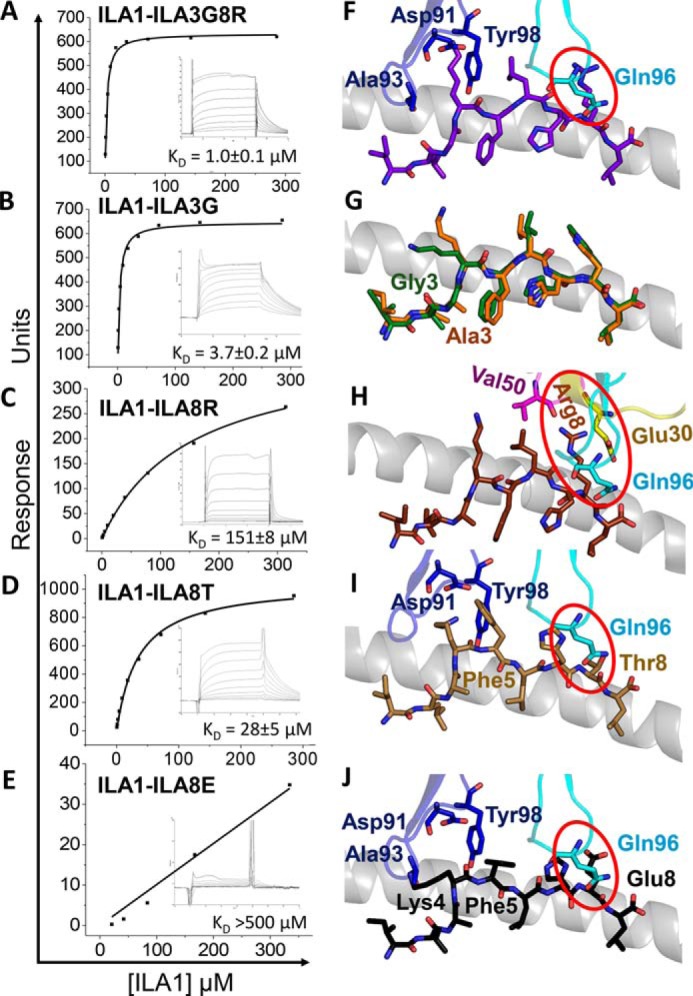
**Equilibrium binding analysis and structural modeling of the ILA1 TCR interaction with the APLs.** Binding affinity of the ILA1 TCR interaction with different APLs at 25 °C is shown. Eight to ten serial dilutions of the ILA1 TCR were injected over A2-ILA8R and A2-ILA3G8R, and representative data from three independent experiments are plotted after deducting binding to a control sample (HLA-A*0201-ALWGPDPAAA). The equilibrium binding constants (*K_D_*) were calculated using a non-linear curve fit (*y* = (P_1_*x*)/P_2_ + *x*)). *A*, ILA1-A2-ILA3G8R. *B*, ILA1-A2-ILA3G (reproduced from Ref. 15). *C*, ILA1-A2-ILA8R. *D*, ILA1-A2-ILA8T (reproduced from Ref. 15). *E*, ILA1-A2-ILA8E (reproduced from Ref. 15). The ILA1 TCR was modeled with each of the APL ligands by aligning the uncomplexed APLs with A2-ILA in the ILA1-A2-ILA complex structure (HLA-A*0101 α1 helix shown in *gray* schematic in *F–J*). Potential steric clashes are highlighted in *red circles. F*, A2-ILA3G8R (peptide in *purple*) showing modeled positions of the TCR CDR3α and -β loops (*blue* and *cyan*, respectively). *G*, A2-ILA3G (peptide in *green*) superposed with the A2-ILA peptide (*orange*). *H*, A2-ILA8R (peptide in *brown*) showing modeled positions of the TCR CDR1, -2, and -3 α/β loops (*yellow*, *pink*, and *cyan*, respectively). *I*, A2-ILA8T (peptide in *sand*) showing modeled positions of the TCR CDR3α and -β loops (*blue* and *cyan*, respectively). *J*, ILA1-A2-ILA8E (peptide in *black*) showing modeled positions of the TCR CDR3α and -β loops (*blue* and *cyan*, respectively).

We next explored the binding affinity of the ILA1 TCR for the APLs included in this study using previously published (9, 15, 16) and new data (Fig. 6, *A–E*, and Table 4). Despite the relatively weak affinity between the ILA1 TCR and the natural A2-ILA ligand (*K_D_* = 34 μm), the ILA1 TCR could recognize A2-ILA3G8R and A2-ILA3G with antiviral-like affinities (*K_D_* = 1.0 and 3.7 μm, respectively). Both of these ligands included substitution of peptide residue 3 from Ala to Gly, a substitution that was clearly indicated in our previously published unbiased combinatorial peptide library screening using the ILA1 T-cell clone (9). Structural modeling of the ILA1 TCR with these ligands indicated that interaction with the N-terminal portion of the peptide was likely to be very similar for both ligands (Fig. 6, *F* and *G*). However, for A2-ILA3G8R, a major reorientation of TCR β-chain residue Gln-96 would be required to tolerate Arg at position 8 in the peptide. For both of these ligands, the extra flexibility, afforded at the N terminus of the peptide by the substitution of Gly compared with Ala, may enable the ILA1 TCR to establish enhanced contacts with peptide residue Lys-4. This represents a likely mechanism for the stronger affinity, supported further by the observation that all of the other APLs that did not include Gly at position 3 were bound by ILA1 with weaker affinity compared with the ILA1-A2-ILA interaction (Fig. 6, *C–E*). Structural modeling of ILA1 in complex with A2-ILA8R (Fig. 6*H*) revealed the same potential for steric hindrance between the TCR β-chain residues Glu-30 and Gln-96 but without the compensatory substitution at position 3. The structural rearrangement that would be required for ILA1 to bind to A2-ILA8R (*K_D_* = 151 μm) was reflected by a much weaker affinity compared with ILA1-A2-ILA (*K_D_* = 34 μm). Both A2-ILA8T and A2-ILA8E ligands underwent a conformational transition compared with the other APLs (Fig. 5). Modeling demonstrated that this alteration could lead to a steric conflict between TCR β-chain residue Gln-96 and the C-terminal residues of the peptide (Fig. 6, *I* and *J*). However, the position of peptide residue Phe-5 in A2-ILA8T may allow for compensatory interactions with TCR α-chain residues Asp-91 and Tyr-98, which are not present with A2-ILA8E. Furthermore, the smaller Thr-8 side chain in A2-ILA8T, compared with Glu-8 in A2-ILA-8E, would require smaller modifications in TCR docking. Taken together, these structural distinctions may explain the extremely weak affinity observed for ILA1 binding to A2-ILA8E compared with A2-ILA8T (*K_D_* values >500 μm and 28 μm, respectively).

**TABLE 4:** **ILA1 TCR binding affinity to peptide variants** *K_D_* was calculated from equilibrium binding experiments. n/m, kinetics were too fast to accurately measure.

HLA-A*0201-ILA variant	*k*_on_	*k*_off_	χ^2^ for *k*_on_ and *k*_off_	*K_D_*
	*m*^−*1*^ *s*^−*1*^	*s*^−*1*^		μ*m*
HLA-A*0201-ILGKFLHRL	3 × 10^4^	0.16	4.2	1.0 ± 0.1
HLA-A*0201-ILGKFLHWL (39)	1.6 × 10^4^	0.05	3.6	3.7 ± 0.2
HLA-A*0201-ILGKFLHTL (17)	1.95 × 10^4^	0.05	2.5	2.5 ± 0.5
HLA-A*0201-ILAKFLHYL (39)	n/m	n/m	n/m	22.6 ± 2.1
HLA-A*0201-ILAKFLHWL (20)	4.5 × 10^3^	0.15	4.3	34 ± 2
HLA-A*0201-ILAKFLHTL (39)	2.2 × 10^3^	0.08	1.9	28 ± 5
HLA-A*0201-ILAKFLYWL (39)	n/m	n/m	n/m	82 ± 8
HLA-A*0201-ILALFLHWL (16)	1.7 × 10^3^	0.2	4.2	117 ± 6
HLA-A*0201-ILAKFLHRL	n/m	n/m	n/m	151 ± 8
HLA-A*0201-ILAKYLHWL (17)	1.3 × 10^3^	0.32	3.5	242 ± 20
HLA-A*0201-ILAKFLHEL (39)	n/m	n/m	n/m	>500

##### Quantification of ILA1 TCR Degeneracy

Our previous investigations have demonstrated that even single residue substitutions outside of the two main peptide interaction zones (Lys-4 and Trp-8) could have a substantial impact on the ILA1 TCR-A2-ILA complex, reflected by the different binding affinities and antigen potencies shown here and published previously (9, 15, 16). We next generated a degeneracy curve for the ILA1 TCR using our previously described approach that quantifies TCR cross-reactivity (3).

**FIGURE 7.**
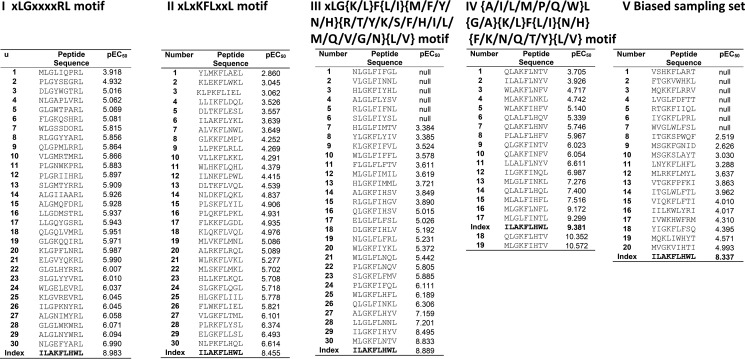
**pEC_50_ values for all peptide ligands tested.** Simultaneous curve fitting was used to estimate functional sensitivity measured as pEC_50_ for peptides sampled from the following sets: *I*, *X*LG*XXXX*RL (set size, 19^5^; 30 peptides sampled at random); *II*, *X*L*X*KFL*XX*L (set size, 19^4^; 30 peptides sampled at random); *III*, *X*LG(K/L)F(L/I)(M/F/Y/N/H)(R/T/Y/K/S/F/H/I/L/M/Q/V/G/N)(L/V) (set size, 10,640; 30 peptides sampled at random); *IV*, (A/I/L/M/P/Q/W)L(G/A)(K/L)F(L/I)(N/H)(F/K/N/Q/T/Y)(L/V); and *V*, replicate of a biased sampling of each set (20 peptides sampled from an effective sample size of 1.5 × 10^7^).

**FIGURE 8.**
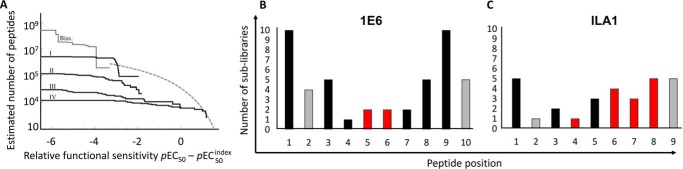
**TCR binding footprint contributes to T-cell cross-reactivity.** *A*, peptide recognition degeneracy for ILA1. The degeneracy curve plots the estimated number of peptides that have a functional sensitivity at least as strong as abscissa. The highest value of abscissa corresponds to inferred functional sensitivity of optimal peptide, whereas the lowest value of abscissa lies 10 orders of magnitude below this optimum. *Bias*, degeneracy curve based on agonist-biased importance sampling. Curves *I–IV* are motif-based, *i.e.* sampled from subsets of the entire peptide universe, and therefore lie below the degeneracy curve. *I*, *X*LG*XXXX*RL (set size, 19^5^; 30 peptides sampled at random); *II*, *X*L*X*KFL*XX*L (set size, 19^4^; 30 peptides sampled at random); *III*, *X*LG(K/L)F(L/I)(M/F/Y/N/H)(R/T/Y/K/S/F/H/I/L/M/Q/V/G/N)(L/V) (set size, 10,640; 30 peptides sampled at random); *IV*, (A/I/L/M/P/Q/W)L(G/A)(K/L)F(L/I)(N/H)(F/K/N/Q/T/Y)(L/V) (set size, 1344; 19 peptides sampled at random); *X* denotes any of the 19 amino acids excluding cysteine. Cross-reactivity of the 1E6 (*B*) and ILA1 (*C*) CD8^+^ T-cell clones was estimated by the number of residues recognized in a combinatorial peptide library screen generating responses over >0.5 ng/ml MIP-1β. *Bars* that represent positions in the peptide that are anchor residues are colored *gray. Bars* that represent residues that are main contact sites for each respective TCR are colored *red*.

Combinatorial peptide library scan data were used to design four different motif-restricted peptide sets (I, *X*LG*XXXX*RL (total set size, 19^5^); II, *X*L*X*KFL*XX*L (total set size, 19^4^); III, *X*LG(K/L)F(L/I)(M/F/Y/N/H)(R/T/Y/K/S/F/H/I/L/M/Q/V/G/N)(L/V) (total set size, 10,640); and IV, (A/I/L/M/P/Q/W)L(G/A)(K/L)F(L/I)(N/H)(F/K/N/Q/T/Y)(L/V) (total set size, 1344) where *X* denotes any of the 19 proteogenic amino acids excluding cysteine). Between 19 and 30 peptides were sampled at random from each of these motif-restricted peptides cohorts. In addition, we performed importance sampling where 20 peptides were sampled from an effective sample size of 1.5 × 10^7^. The pEC_50_ for each peptide was estimated by simultaneous curve fitting (Fig. 7), and these values were used to construct a degeneracy curve for the ILA1 TCR (Fig. 8*A*). These analyses indicated that ILA1 could recognize ∼2 × 10^3^ peptides with a functional sensitivity at least as high as 110 the functional sensitivity of the optimal agonist. At 2 orders of magnitude from the optimum (*i.e.* peptides ranging from 1100 of the optimal agonist to the optimal agonist) ∼4 × 10^4^ peptides could be recognized by the ILA1 T-cell clone.

This analysis suggests that the ILA1 TCR can cross-react with a diverse peptide universe. Although smaller than the estimated number of peptides recognized by the 1E6 T-cell clone (∼10^6^), it should be noted that 1E6 recognizes a 10-mer peptide, whereas ILA1 recognizes a 9-mer. Thus, the peptide universe under consideration for 1E6 is 20 times larger than that for ILA1. Although it is unknown how this difference in peptide length affects the comparison between the two degeneracy profiles, this difference may partly explain why ILA1 appears to be less cross-reactive than 1E6. This analysis also demonstrated a different pattern of cross-reactivity between the 1E6 and ILA1 T-cell clones that was consistent with the respective binding footprints of their TCRs. The 1E6 T-cell clone could recognize a large number of sublibraries (46 in total) outside of the central binding zone (residues 4–6) (Fig. 8*B*). In contrast, the ILA1 T-cell clone was generally more sensitive across the peptide backbone, reflecting a more globally coordinated interaction between the ILA1 TCR and the antigenic peptide (Fig. 8*C*). Taken together, these results are broadly consistent with the idea that different TCR binding footprints (*i.e.* TCRs that focus on a minimal peptide motif compared with TCRs that make contacts across the peptide backbone) can enable T-cell cross-reactivity, adding further support to the general idea that T-cells must be cross-reactive to fully protect us against a highly variable pathogen universe (for a review, see Ref. 8).

## Discussion

To mount effective immune responses in the face of a diverse antigenic milieu using a limited set of TCRs (estimated ∼25 million distinct clonotypes in an individual), each T-cell must be able to interact productively with a vast array of different antigens (2, 8). Indeed, recent experimental evidence supports this notion, including our own study demonstrating that a single T-cell clone can recognize over one million different peptides at physiologically relevant concentrations (3). However, structural investigations of TCR-pMHC interactions have demonstrated that the TCR can establish a distinct and highly specific interaction with both peptide and MHC. In keeping with such specificity of binding requirements, even single mutations at key residues in the TCR, peptide, or MHC that are involved in the binding interface have been shown to abrogate antigen recognition (25–29). The importance of T-cell cross-reactivity is manifest not only in effective immune surveillance (2, 8) but also in autoreactivity (30) and the design of therapeutics (10, 11). Structural rules must therefore exist that allow cross-reactivity to take place; here, we report early steps toward deconstructing these rules.

We investigated a TCR isolated from ILA1, a well characterized HLA-A*0201-restricted, telomerase-specific, CD8^+^ T-cell clone. Our previous work has shown that ILA1 cross-reacts with an array of APLs with different potencies, tuned by the CD8 co-receptor, and that antigen “potency” generally correlates directly with the affinity of TCR binding (9, 15). Here, we solved the complex structure of the ILA1 TCR with the natural index ligand (A2-ILA) and used the structures of a number of unligated APLs to model the mode of APL recognition and determine the structural basis for ILA1 cross-reactivity.

We have demonstrated previously that the ILA1 T-cell clone is particularly sensitive to APLs with modifications at peptide residues 3 and 8 (15). The unligated structures of five APLs with alterations in these positions demonstrated that the overall conformation of the Cα peptide backbone could be altered by introduction of Thr or Glu at position 8, possibly explaining the weaker binding affinity between ILA1 and A2-ILA8R and between ILA1 and A2-ILA8E. In contrast, substitution of Ala to Gly at peptide residue 3 enhanced recognition, and Gly at this position was strongly recognized in combinatorial peptide library screens (9). Our structural analysis indicated that the surmised extra flexibility afforded to the N terminus of the peptide mediated by substitution at position 3 with Gly would likely enable more favorable interactions with Lys-4, which made a network of contacts with the ILA1 TCR through a “ball-and-socket”-like interaction. In fact, substitution at position 3 with Gly could override the negative impact of modifications to peptide residue 8, revealed by the enhanced binding affinity of the ILA1-A2-ILA3G8R interaction (*K_D_* = 1.0 μm) compared with the index peptide (*K_D_* = 34 μm) and ILA8R (*K_D_* = 151 μm). Our structural analysis demonstrated that, again, even single peptide substitutions outside of the main interaction interface could have a substantial impact on TCR binding affinity and T-cell antigen potency, consistent with our previous data (9, 15, 16). These observations add further evidence to our recent findings (19, 31–34) that peptide presentation by MHCI can be dynamic and difficult to predict.

Recent reports have demonstrated that TCRs using focused TCR-peptide binding can be highly cross-reactive (6, 36). However, this binding footprint is not representative of most TCRs described in the literature. Indeed, on average, TCR-peptide binding is spread out over ∼60% of the peptide backbone for MHCI-restricted epitopes, often including contacts with both the N- and C-terminal regions of the peptide (1, 37). This interconnected binding network between the TCR and the peptide may not allow a high degree of cross-reactivity because most peptide modifications could impact binding. Unlike the focused TCR-peptide binding utilized by the 1E6 (6) and 42F3 (38) TCRs, the ILA1 TCR utilized a more representative binding footprint. This was reflected by a larger buried surface area value (2540 Å for ILA1 compared with 1640 Å for 1E6) and a binding motif that included contacts spread out over peptide residues 4–8. Thus, we explored the consequences of the ILA1 TCR binding footprint on ILA1 T-cell cross-reactivity using our previously published methodology (3). Despite the broader peptide contact zone utilized by the ILA1 TCR, the ILA1 T-cell clone was still able to recognize ∼4 × 10^4^ peptides with equal or greater sensitivity compared with the index peptide and many more at lower potency. These data suggest that although the TCR binding footprint is very likely to tune T-cell cross-reactivity to some degree the ability of T-cells to recognize a vast array of different peptides is likely to be commonplace.

In summary, we demonstrate that the interaction between a TCR from a human CD8^+^ T-cell clone that recognizes a peptide sequence from an important tumor antigen contacts the peptide at spatially distant sites along the peptide backbone. The affinity of this TCR can be tuned by various peptide modifications through both direct and indirect effects, demonstrating the dynamic nature of the interaction among TCR, peptide, and MHC. Even though the ILA1 T-cell clone was sensitive to modifications along the peptide backbone, consistent with its broad binding interface, it was still able to cross-react with a vast array of different peptides. These data demonstrate that focused TCR-peptide binding is not a requirement for T-cell degeneracy. Indeed, a broader binding footprint, as observed in most TCR-pMHC structures reported to date, is also likely to facilitate T-cell cross-reactivity. These results have important implications for immune surveillance, *i.e.* how a limited set of TCRs can recognize all potential antigens variants, and the complex mechanisms that may lead to autoreactivity mediated by molecular mimicry.

## Experimental Procedures

### 

#### 

##### T-cells and Target Cells

The ILA1 CD8^+^ T-cell clone is specific for the HLA-A*0201-restricted human telomerase reverse transcriptase-derived epitope ILAKFLHWL (residues 540–548) (39), and the 1E6 T-cell clone is specific for the human leukocyte antigen HLA-A*0201-restricted autoantigen preproinsulin epitope ALWGPDPAAA (residues 15–24) (40). CD8^+^ T-cell clones were maintained in RPMI 1640 medium (Life Technologies) containing 100 units/ml penicillin (Life Technologies), 100 mg/ml streptomycin (Life Technologies), 2 mm l-glutamine (Life Technologies), and 10% heat-inactivated FCS (Life Technologies) (R10) supplemented with 2.5% Cellkines (Helvetica Healthcare, Geneva, Switzerland), 200 IU/ml IL-2 (PeproTech, Rocky Hill, NJ), and 25 ng/ml IL-15 (PeproTech). Hmy.2 C1R B-cells expressing full-length HLA-A*0201 were generated as described previously (41).

##### Protein Expression, Refolding, and Purification

The ILA1 TCR, HLA-A*0201 α-chain, and human β_2_-microglobulin chain sequences were generated as described previously (20) and cloned into separate pGMT7 expression plasmids under the control of the T7 promoter. The ILA1 TCR and HLA-A*0201 in complex with various different peptide variants (as indicated) were refolded and purified as described previously (14). Biotinylated pMHCI was prepared as described previously (42).

##### pMHC Stability Assays

Thermal stability of the HLA-A*0201-peptide complexes was assessed by circular dichroism spectroscopy, monitoring the change in ellipticities at 218 nm upon heating as described (31). Briefly, samples were prepared in PBS at a concentration of ∼3 μm and measured in 0.1-cm quartz cells. Melting curves were analyzed assuming a two-state trimer-to-monomer transition from the native to unfolded conformation and fitted as described (43). As all protein complexes aggregated to various degrees upon unfolding, the ellipticity of the unfolded state was set as a constant of −1.35 m^−1^ cm^−1^ (44).

##### Surface Plasmon Resonance Analysis

Binding analysis was performed in duplicate using a BIAcoreT200^TM^ equipped with a CM5 sensor chip as described previously (45). Approximately 200–500 response units of HLA-A*0201-ILGKFLHRL or HLA-A*0201-ILAKFLHRL peptide complex was attached to the CM5 sensor chip at a slow flow rate of 10 μl/min to ensure uniform distribution on the chip surface. HLA-A*0201-ILAKFLHWL was used as a positive control as the binding affinity with the ILA1 TCR has been published previously (15, 20). The ILA1 TCR was purified and concentrated to ∼300 μm on the same day of surface plasmon resonance analysis. For equilibrium analysis, 10 serial dilutions were prepared in duplicate for each sample and injected over the relevant sensor chips at 25 °C. TCR was injected over the chip surface using kinetic injections at a flow rate of 45 μl/min using HLA-A*0201-ALWGPDPAAA as a negative control surface on flow cell 1. Results were analyzed using BIAevaluation^TM^ 3.1, Excel, and Origin 6.0 software. The equilibrium dissociation constant (*K_D_*) values were calculated assuming a 1:1 interaction by plotting specific equilibrium binding responses against protein concentrations followed by non-linear least square fitting of the Langmuir binding equation. For kinetics analysis, the *k*_on_ and *k*_off_ values were calculated assuming 1:1 Langmuir binding, and the data were analyzed using a global fit algorithm (BIAevaluation 3.1).

##### Crystal Structure Determination

All protein crystals were grown at 18 °C by vapor diffusion via the sitting drop technique. 200 nl of each pMHCI (10 mg/ml) in crystallization buffer (10 mm Tris, pH 8.1, and 10 mm NaCl) was added to 200 nl of reservoir solution. ILA1-HLA-A*0201-ILAKFLHWL (ILA1-A2-ILA) and HLA-A*0201-ILAKFLHTL (A2-ILA8T) crystals were grown in 0.2 m ammonium sulfate, 0.1 m HEPES, pH 7, and 20% PEG 8000 (46); HLA-A*0201-ILGKFLHRL (A2-ILA3G8R) crystals were grown in 0.2 m ammonium sulfate, 0.1 m Tris, pH 7.5, and 25% PEG 8000 (46); HLA-A*0201-ILGKFLHWL (A2-ILA3G) crystals were grown in 0.2 m ammonium sulfate, 0.1 m MES, pH 7, and 15% PEG 8000; and HLA-A*0201-ILAKFLHEL (A2-ILA8E) crystals were grown in 0.2 m ammonium sulfate, 0.1 m MES, pH 7, and 25% PEG 8000 (46). Crystallization screens were conducted using an Art-Robbins Phoenix dispensing robot (Alpha Biotech Ltd., UK), and data were collected at 100 K at the Diamond Light Source, Oxfordshire, UK, at a wavelength of 0.98 Å using an Area Detector Systems Corp. Q315 charge-coupled device detector. Reflection intensities were estimated using XIA2 (47), and the data were analyzed with SCALA and the CCP4 package (48). Structures were solved with molecular replacement using Phaser (49). Sequences were adjusted with Coot (50), and the models were refined with REFMAC5. Graphical representations were prepared with PyMOL (35). The reflection data and final model coordinates were deposited with the Protein Data Bank under codes 5MEN (ILA1-A2-ILA), 5MEO (A2-ILA3G8R), 5MEP (A2-ILA3G), 5MEQ (A2-ILA8T), and 5MER (A2-ILA8E).

##### CD8^+^ T-cell Effector Function Assays: MIP1β ELISA

6 × 10^4^ C1R-A2 cells were incubated with peptide at various concentrations in duplicate for 2 h at 37 °C. Subsequently, 3 × 10^4^ ILA1 CD8^+^ T-cells were added, and the assay was incubated overnight at 37 °C. The supernatant was harvested and assayed for MIP1β by ELISA according to the manufacturer's instructions (R&D Systems). Functional sensitivity of individual peptides was expressed as the pEC_50_ of each peptide, which is defined as −1 × the base 10 logarithm (p) of the 50% efficacy concentration (EC_50_).

##### Quantification of ILA1 TCR Degeneracy

The degeneracy of the ILA1 TCR was estimated as described previously (3). Briefly, the degeneracy at ω, defined as the number of peptides whose functional sensitivity is at least as large as ω, was estimated directly using importance sampling based on the combinatorial peptide library scan and bounded below by sampling from motif-based subsets of the peptide universe. The degeneracy is reported by plotting this quantity as a function of ω where the functional sensitivity ω was scaled relative to a clone-specific reference peptide (the “index”).

## Author Contributions

H. A. v. d. B., A. L., M. D. C., K. B., J. E.-M., J. J. M., A. M. B., G. D., A. J. S., A. W., A. F., M. C., B. L., P. J. R., L. W., and D. K. C. performed experiments and analyzed the data. A. K. S., L. W., and D. K. C. wrote the manuscript. A. K. S., L. W., H. A. v. d. B., and D. K. C. conceived and directed the study. A. K. S., L. W., and D. K. C. funded the study. All authors contributed to discussions.

## Footnotes

5 The abbreviations used are:

pMHC: peptide-major histocompatibility complex
TCR: T-cell receptor
CDR: complementarity-determining region
APL: altered peptide ligand
A2-ILA: HLA-A*0201-ILAKFLHWL
EC_50_: 50% efficacy concentration.
